# Numerical Investigations of Perforated CFRP Z-Cross-Section Profiles, under Axial Compression

**DOI:** 10.3390/ma15196874

**Published:** 2022-10-03

**Authors:** Katarzyna Falkowicz

**Affiliations:** Department of Machine Design and Mechatronics, Faculty of Mechanical Engineering, Lublin University of Technology, Nadbystrzycka 36, 20-618 Lublin, Poland; k.falkowicz@pollub.pl

**Keywords:** profiles with cut-out, critical force, buckling, postbuckling, FEM

## Abstract

Thin-walled elements, thanks to their good properties, are increasingly used in structural applications, especially in the aircraft and building industries. These kinds of structures are often perforated for reducing weight and to ease servicing and maintenance operations, e.g., in aircraft wing ribs. These perforations cause a redistribution of stresses in the element which may change the ultimate strength of the structure and their elastic stiffness. The buckling behaviour of structural members with perforations is significantly influenced by the size, location, shape and number of perforations. Therefore, it is necessary to investigate the influence of these kinds of cut-out parameters on thin-walled structure buckling and postbuckling behaviour. This study investigated numerically the buckling and postbuckling behaviour of thin-walled perforated composite profiles with a Z-cross-section subjected to compression load. Numerical calculations were performed using the finite element method in the ABAQUS^®^ program. The study investigated the effect of localisation and geometric parameters of cut-outs on the buckling load, postbuckling equilibrium path and failure load. Moreover, the perforated profiles were compared with a profile without cut-outs, which were experimentally tested in previous research. Results showed that the perforated profiles with a Z-cross-section do not lose their stability in the post-critical range. What is more, a well-chosen arrangement of the holes may prevent the mechanical properties from deteriorating.

## 1. Introduction

The industry is constantly evolving, which influences a growing needs for advanced composite design technologies, which also have a significant role in industries such as aviation, automotive and aerospace. The realisation of these goals is associated with continuous work in improving the design, production technology and selection of materials. The industry is increasingly moving away from classical engineering materials, i.e., metals, which are being replaced by composites. Thanks to their good properties, such as high strength to weight ratio, excellent corrosion resistance and very good fatigue strength, these materials are becoming more and more popular in aviation [1,2], construction [3,4] and medicine. The load-bearing capacity of these structures depends mainly on the number of used laminate layers, the direction of fibres in individual layers and the properties of the material. A feature of structural elements made of fibrous composites is also a large margin of structural load capacity, i.e., the ability to work after loss of stability, sometimes reaching 2–3 times the value of the critical load. This feature distinguishes these materials from metal structural elements, in which permanent deformations achieve approx. 150% of the critical load value. Moreover, composite structures generally fail as a result of brittle cracking of the material. This essentially eliminates the risk of local plasticization of critical areas of the load-bearing structure, which can lead to the rapid failure of the structure. The high reserve of the load-bearing capacity of composite elements increases the operational safety of the structure, which, even with the first symptoms of composite structure damage, maintains high stiffness, practically until achieved failure load. These features of fibrous composites, combined with the low density of the material, create great possibilities for their application in many areas, including the aviation, automotive and space industries, where design solutions based on thin-walled plate and shell elements are dominant, and where minimization of weight is highly desirable.

Thin-walled structures are often perforated for reducing weight and to ease servicing and maintenance operations, e.g., in aircraft wing ribs. These perforations cause a redistribution of stresses in the member that may change the ultimate strength of the structural member and the elastic stiffness.

The buckling behaviour of structural members with perforations is significantly influenced by the size, location, shape and number of perforations. Therefore, it is necessary to investigate the influence of these kinds of cut-out parameters on thin-walled structure buckling and postbuckling behaviour.

For the correct understanding of the mentioned problem and buckling phenomenon, the characteristics of composite structure stability and plate elements with cut-outs are necessary.

The problem of stability of compressed thin-walled structures with the finite element method was analysed by Teter and Kolakowski [5], Debski (ed.) [6], Paszkiewicz and Kubiak [7] and Bazant and Cedolin [8]. Based on linear numerical procedures, the authors determined the values of the critical load and the lowest eigenform of the considered thin-walled structures. Post-critical studies with nonlinear analysis were presented in the publications of Kopecki [9], Thompson et al. [10] and Wysmulski [11]. The authors took under consideration nonlinear relationships between displacements and deformations. This let it possible to determine the post-critical equilibrium paths, which determine the relationships between the static parameters corresponding to the load of structure and the geometric parameters determining the displacement of its nodes. The results of research related to the analysis of the post-critical state of composite structures, working under compressive load, were presented, among others, by: Banat et al. [12], Dębski et al. [13], Debski et al. [14] and Rozylo [15]. More examples of using FEM to solve the problems of linear and nonlinear stability of composite structures can be found in the studies of Camanho and Davila [16], Alfano and Crisfield [17], Kreja [18] and Mani [19], among others. The authors of [20,21,22] investigated experimentally the overall stability of composite members under compression loading, with three different cross-sections.

On the other hand, buckling analysis of plates with different cut-out shapes has been studied by many researchers. The research of the laminated plates stability, with cut-outs, was carried out mainly on rectangular plates [23,24,25]. On their basis, it was found that the buckling susceptibility of rectangular plates depends on the boundary conditions, the arrangement of layers [26,27] or the rounding of cut-out radius [23,26]. Akbulut and Sayman [28] performed a buckling analysis of rectangular composite plates with a central square hole. Komur et al. [29] studied the buckling of composite square plates with an elliptical cut-out. They showed that designers should avoid large, elliptical holes in composite plates if they want to prevent buckling at low pressures. Ouinas and Achour [30] analysed the buckling of square plates with an elliptical hole; they studied the influence of the size and location of the hole, as well as the influence of asymmetric layers layouts on the critical force.

There have also been investigations on aluminium alloy members with perforations [31]. The authors investigated the buckling behaviour of aluminium alloy square hollow sections (SHS) with a circular hole under web crippling. Paper [32] presents the results of numerical analysis of compressed thin-walled Z-profiles weakened by holes with variable geometrical parameters, but made of constructional steel. In [33], the authors made some analysis of H-shaped high-strength steel beams with perforated web. In this study the effects of the diameter and number of holes in the web on the flexural strength and failure mode of H-shaped high-strength steel beams were evaluated. Paper [34] presents experimental and numerical investigations on slender panels with holes under symmetrical localised loads. Parameters such as the geometry of the web plate, the position of the hole and the plate slenderness are found by authors as those most influencing the post-critical behaviour. Moreover, in study [35], where aluminium alloys were used as a subject of the experiment, the authors tested the geometry and position of holes. In this paper, three types of shape, circular, hexagonal and rectangular, were chosen. The authors also took the space between holes and the size of holes as the parameters for their study. Similar analyses, but on GFRP composite profiles, were performed in work [36].

From the above-mentioned literature, buckling analysis of composite plates with different cut-out shapes, such as circular holes, elliptical holes, square or rectangular holes, has been the subject of much research. Results presented in the related literature indicate that the buckling behaviour of composite plates is affected by cut-out shape, cut-out size and cut-out orientation. However, the combination of variables considered during previous studies is still limited, and research is connected mainly to plate elements or profiles with different kinds of cross-section, but made of classical materials such as steel and aluminium. What is more, in the literature is hard to find publications where the tested composite profiles are weakened by cut-outs. This area is still not well-tested. Moreover, in previous articles [37,38,39,40], the authors researched composite plate elements weakened by cut-out and composite profiles with different kinds of cross-section [41,42,43], but without cut-outs. Therefore, this has ignited author motivation, together with the papers by [35,36], for the analysis presented in this study, and hence contributes towards the knowledge on thin-walled composite structures with cut-outs.

This paper includes the linear and nonlinear analysis of compressed, thin-walled, perforated profiles with a Z-cross-section, which are used in industry. It should be underlined here that the tested profiles are made of composite material, and previous research has been carried out on perforated profiles made from classic materials. It should also be mentioned here that there are no specific standards for the perforation of composite profiles. The research included linear and nonlinear numerical analysis using the finite element method, which has been widely used in recent years [44,45]. In addition, perforated profiles were compared with profiles without cut-outs, which had been previously experimentally tested. The study assessed the impact of the arrangement of the openings on the profile web and shelves, as well as the influence of holes’ geometric parameters and their shape on the stability and work of profiles in the post-critical range.

## 2. Subject of Study

The subjects of the research were thin-walled, perforated Z-cross-section composite profiles subjected to compression. The columns were analysed in their critical and post-critical states. The tested elements were typical thin-walled structures consisting of perpendicular walls, which were flat plate elements, connected along the longer edges.

**Figure 1:**
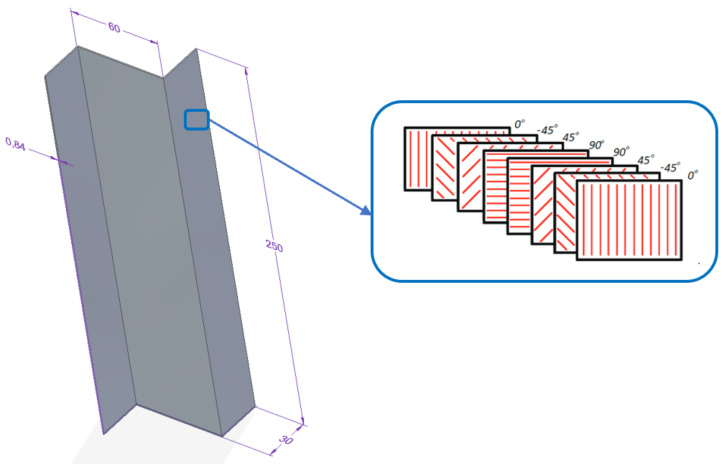
Geometric dimensions of the analysed Z-profile.

Figure 1 shows the general model of the Z-profile which was used in the analysis. The geometric dimensions of each model are constant; they differ only the location and size of the cut-outs. All profiles were modelled in the classical arrangement of fibres. The selected composite configuration consisted of 8 layers. A single layer of the laminate equalled 0.105 mm of thickness, while the thickness of the profile wall equalled 0.84 mm. The structure of the laminate was designed with using the *Laup-Ply* modelling technique, which enables mapping the configuration of the composite layer arrangement according to the thickness of the finite element. In the developed models, a model of an orthotropic material in a plane stress state was defined.

The profile was modelled using the Carbon Fibre Reinforced Polymer (CFRP) material—a composite reinforced with carbon fibre with a polymer matrix. The main mechanical properties are presented in Table 1 (the properties of the composite material determined based on ISO standards: static tensile test—ISO 527, compression test—ISO 14126 and shear test—ISO 14129).

**Figure 2:**
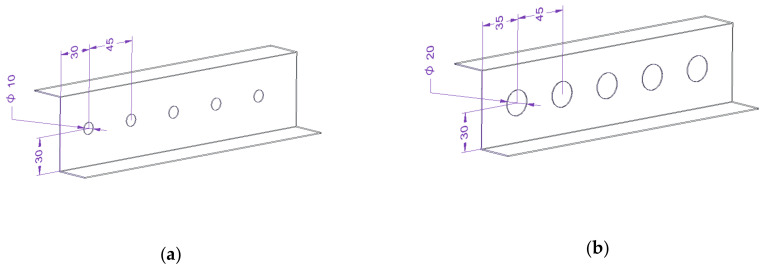

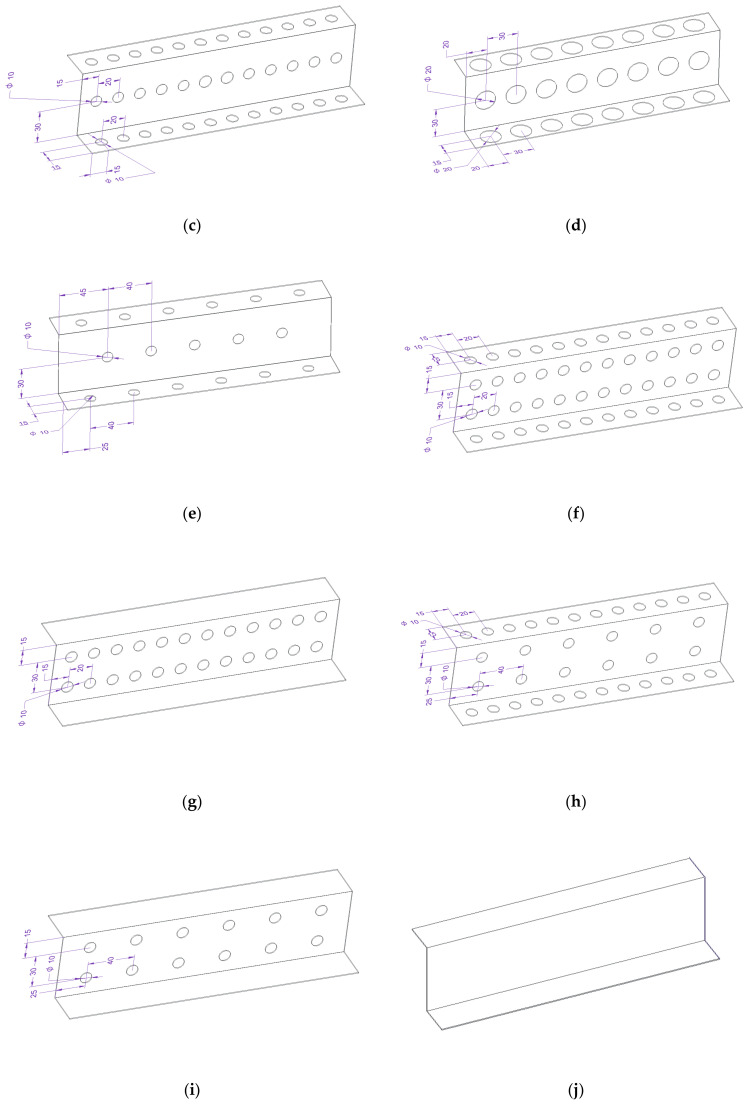
Z-profiles in various cut-out configurations: (**a**) Z1, (**b**) Z2, (**c**) Z3, (**d**) Z4, (**e**) Z5, (**f**) Z6, (**g**) Z7, (**h**) Z8, (**i**) Z9, (**j**) Z0.

Ten profiles were selected for the analysis, nine with round cut-outs of various diameters arranged in a regular or chess configuration, and one without cut-out, as the base profile (Figure 2). Additionally, in one case the openings were located on the web and on the shelves, and in the other case—only on the web. The profiles were numbered from Z0 to Z9. The arrangement of the cut-outs in the analysed profiles was partially mapped on the basis of perforated elements with the same cross-section, which are available on the market, but made of classic materials.

## 3. Research Methodology

**Figure 3:**
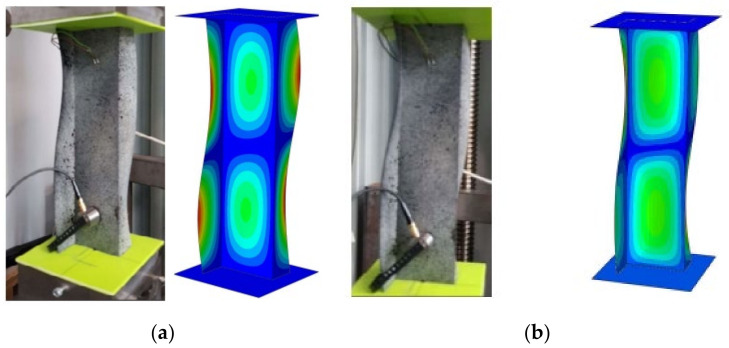
(**a**) Form of buckling, (**b**) form of post-critical deformation.

The author and others, in their previous works, investigated the stability of composite profiles with different kinds of cross-sections, as well with Z-cross-section, which were subjected under compressive load [21]. Physical models of composite columns were made from CFRP (Carbon Fibre Reinforced Polymer) from unidirectional carbon–epoxy prepreg (EP137–CR527/100–35) by autoclave technique. The profile thickness in the experiment was consistent with the profile thickness in numerical analysis. The buckling forms obtained by numerical simulations were positively verified by experimental tests. The buckling and postcritical deformation forms are shown in Figure 3. Moreover, in Table 2, the differences between buckling load from numerical results and the experimental test were presented.

In reference to the above results, the analysed continuous profiles became a reference point for research presented in this paper. This work focuses on the behaviour of perforated Z-cross-section profile and on the evaluation of the impact of geometric parameters of cut-outs and their distribution, and on the compression strength of composite profiles. This paper does not concern the in-depth presentation of the experimental results; only the numerical analysis is presented, but this is the start for further experimental research.

### Numerical Analysis

The numerical analysis was performed in Abaqus program with using FEM. Numerical calculations were carried out in two stages. The first stage was the analysis of the critical state of the structure using linear buckling analysis, which enabled the determination of the critical loads of the compressed element and the corresponding buckling forms. The second stage of the calculations was to solve the problem of nonlinear stability, in which the calculations were carried out on a model with an initiated geometric imperfection corresponding to the lower buckling form of the structure. The value of the initial amplitude was 0.05 mm. The calculations were carried out in accordance with the adopted Tsai-Wu criterion, until failure of the first layer.

The perforated profiles were modelled and analysed in ABAQUS software. The plate structure used in the numerical analysis was discretized using 8-node shell elements (S8R) of the second order with reduced integration. The structure of the composite material was defined with respect to thickness of the finite element. The modelling of the laminate structure was based on the Layup-Ply technique, thanks to which the configuration of the laminate layers was mapped to the thickness of the profile.

**Figure 4:**
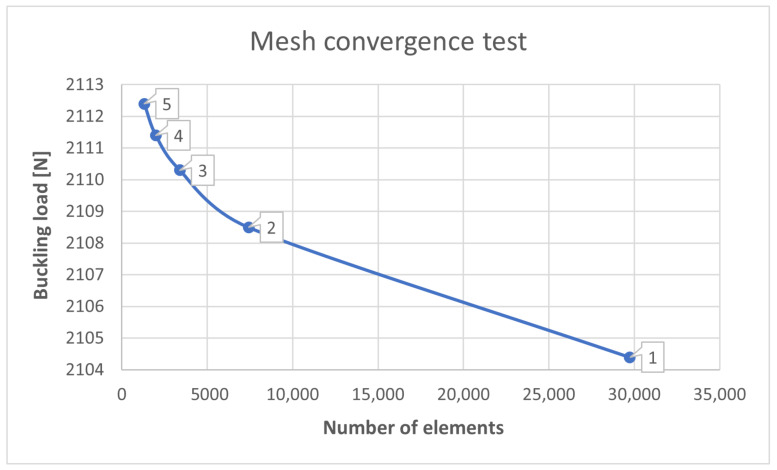
The convergence test results.

To investigate the effect of meshing size, in this study we tested five different element sizes: from 1 mm to 5 mm, for chosen profile type. The mesh convergence was established by increased the mesh density in each part of the model. It was observed that there were no considerable changes in load response between 1 mm and 5 mm element size (Figure 4), but the processing time was considerable and any increment in mesh density is unnecessary, as shown in Figure 4. Therefore, an element size of 4 mm was used in subsequent analysis.

**Figure 5:**
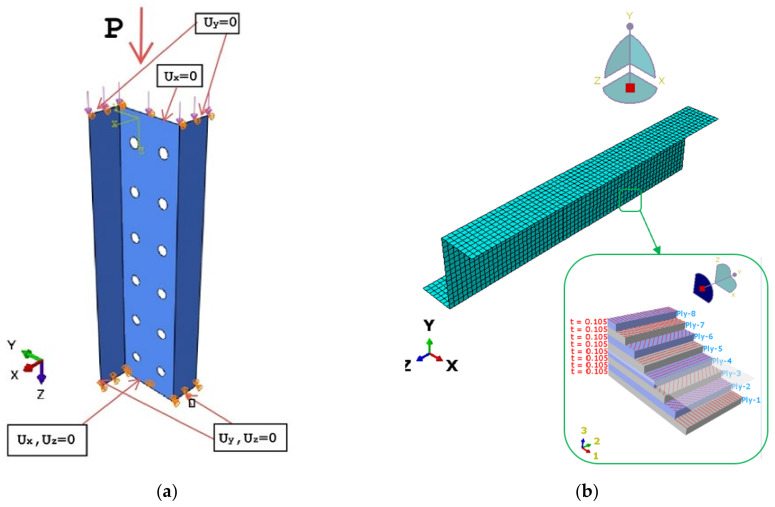
(**a**) Boundary conditions, (**b**) discrete model.

The boundary conditions formulated for the numerical model ensured articulated support of the compressed composite columns—Figure 5a. In Figure 5b presented discrete model of the structure. The boundary conditions were ensured by applying zero displacements to the nodes located on the edges of the lower and upper sections of the column, perpendicularly to the plane of each wall (displacements Ux = 0 and Uy = 0). In addition, the nodes from the bottom end of the column were blocked to prevent vertical displacement (Uz = 0), while the nodes belonging to the edge of the top end of the column were described by the same displacement Uz = const via coupling the displacements relative to the axis of the column. The numerical model was subjected to load applied to the edge of the upper section of the column, ensuring that the column was under uniform compression in the axial direction.

## 4. Results and Discussion

The conducted analysis concerned ten thin-walled composite profiles with a Z-cross-section—including nine perforated profiles with different cut-outs locations and one profile without cut-outs. Thanks to this, it was possible to carry out a comparative analysis of the impact and arrangement of cut-outs on the results of the critical and post-critical state of the columns until the failure of the first layer of the laminate. The profile without cut-outs was used as a reference point to determine the influence of perforation on the mechanical properties of the analysed columns.

**Figure 6:**
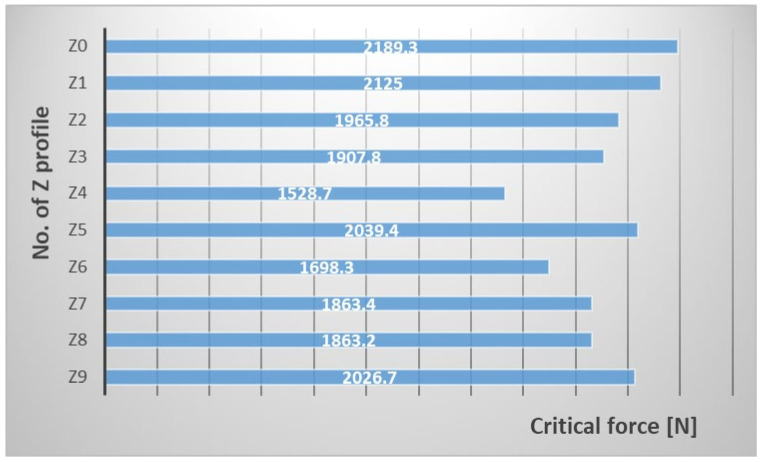
A graph showing the values of the critical force.

FEM numerical analysis Fallowed to assess the critical state of the perforated profiles subjected to compression. The values of the critical forces and the corresponding lowest forms of stability loss were compared. In the analysed profiles, the structure loss of stability was characterized by local buckling, characterized by two half-waves in the longitudinal direction of the column. Table 3 presents the numerically determined critical load values for all analysed cases. Moreover, for better comparison, results in graph form in Figure 6 are presented.

The presented results show that the value of the critical force for perforated composite profiles is lower than for a profile without cut-outs. What is more, the location and size of the holes significantly effect the critical value. The biggest difference, in comparison with the model without cut-out, was observed for the Z4 profile and was 660.6 N. This model is characterized by holes of 20 mm of diameter in the axis of the web and the axis of the shelves. Whereas, Z1 profile had the closest value of the critical force to the non-perforated laminate. During the analysis, we obtained a value lower by 64.3 N.

From the comparative analysis of the Z2 and Z3 profile, it can be concluded that the diameter of the holes has a greater influence on the results than their number. The first one had only 5 holes with a diameter of 20 mm in the axis of the web and the second one had 36 symmetrically arranged holes along the shelves and the web, but with a smaller diameter. Despite a much larger number of holes, the difference in the critical force is 58 N.

The presented results show that despite of the decrease in rigidity, each of the profiles still shows good strength properties. Z4 profile was the least rigid from all tested profiles. The critical force was approx. 30% lower compared to the profile without cut-outs. It is also worth paying attention for the Z3 and Z6 profiles, where the location of the cut-outs and their diameters are practically identical. The difference relates to the web where the cut-outs for the Z3 model were made in one row and for the Z6 model in two rows. The difference in the value of the critical force for these two profiles equal 267.5 N. It can be concluded that the additional row of cut-outs significantly weakened the laminate.

The linear analysis excluded that the perforation of the thin-walled Z-section profiles quickly destroy the structure and the composite loses its properties. The used composite configuration has proven effective in profiles with cut-outs and maintains the original properties of the composite. However, it can be noticed that the value of the critical force has influence on the appropriate selection of the geometric parameters of the cut-outs and their arrangement.

**Figure 7:**
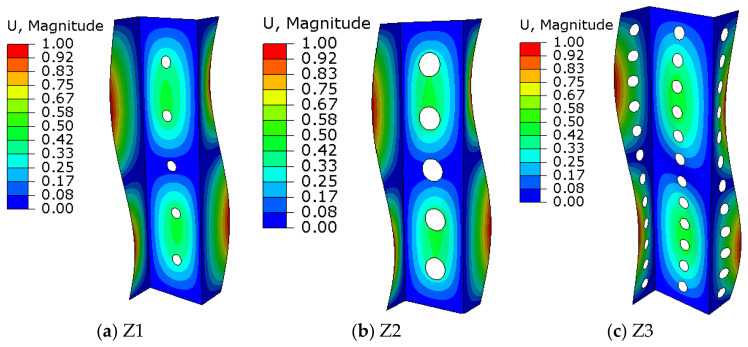
Forms of buckling for exemplary profiles: (**a**) Z1 profile, (**b**) Z2 profile, (**c**) Z3 profile.

In Figure 7 additionally showed the form of buckling for exemplary profiles. For the rest considered cases form of buckling was the same.

During the compression analysis of the Z-profiles, the local buckling of the web and profile walls was characterized by the formation of two half-waves in the longitudinal direction of the column (Figure 7). On the basis of the presented figures, it can be seen that the most heavily loaded were the profile shelves, which underwent the most deformation. In all analysed cases, the highest value of deformation is observed on the shelves. Characteristic places of occurrence of the maximum deformation values for nine profiles are places of half-waves located on opposite sides of the profile—the first and the last half-wave.

**Figure 8:**
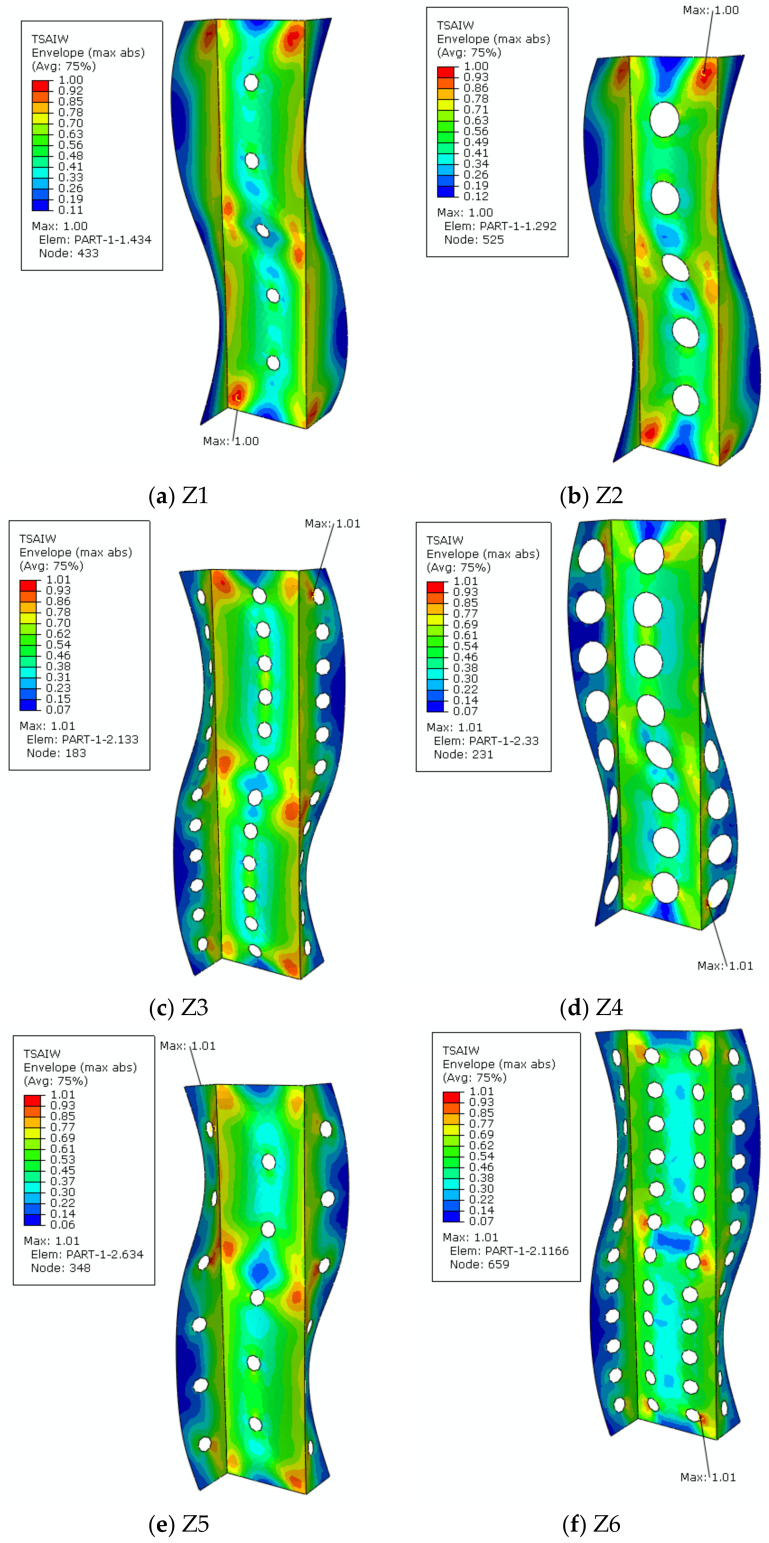

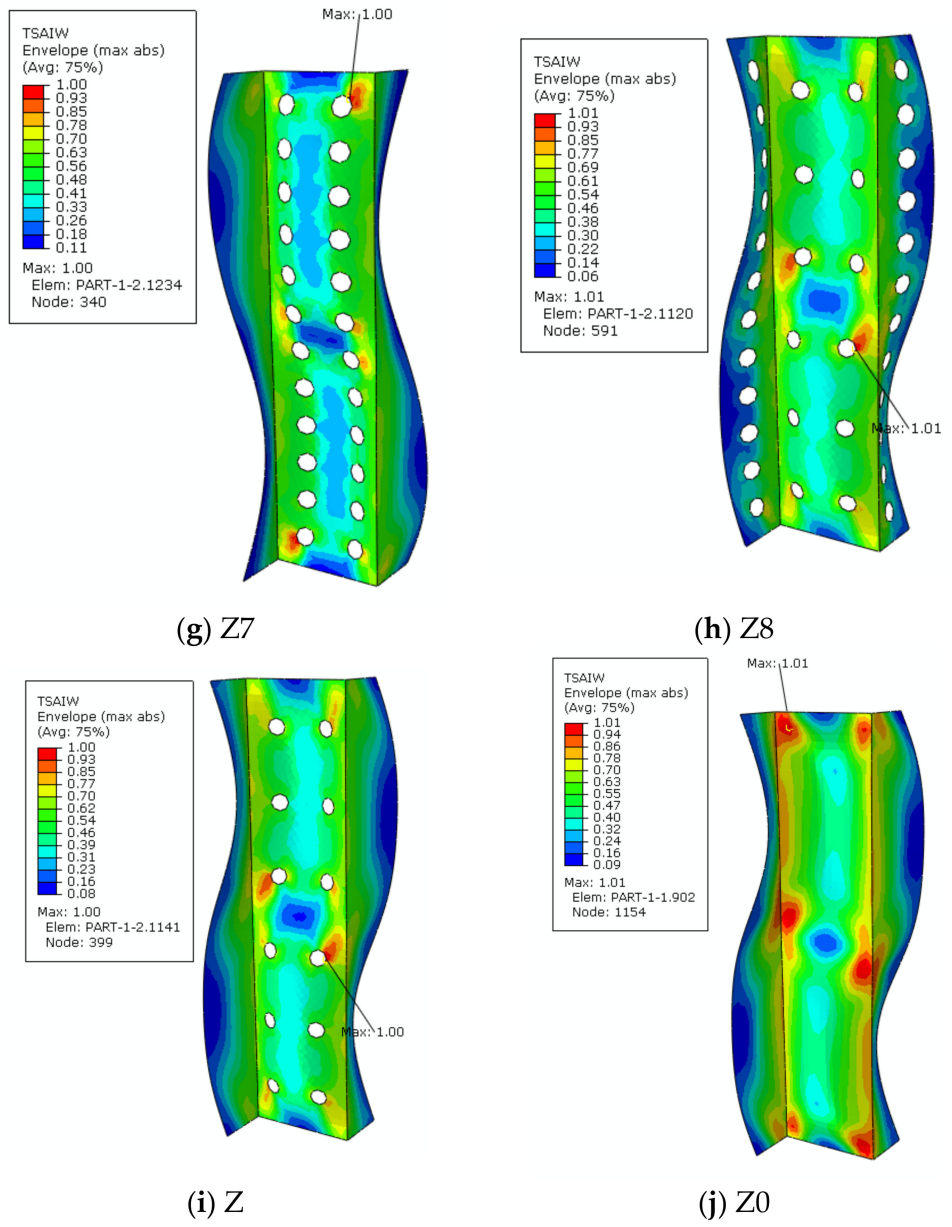
Maps of the Tsai-Wu critical parameter: (**a**) Z1 profile, (**b**) Z2 profile, (**c**) Z3 profile, (**d**) Z4 profile, (**e**) Z5 profile, (**f**) Z6 profile, (**g**) Z7 profile, (**h**) Z8 profile, (**i**) Z9 profile, (**j**) Z0 profile.

Compering the results with the Z-profile without cut-outs (Figure 8j), it can be observed that the cut-outs placed on the web do not have a significant impact on its deformability. No change in the deformation value was noticed in any of the tested profiles after making cut-outs. Profile shelves were always the most vulnerable.

The performed analysis of the critical state of perforated profiles with a Z-cross-section proved a significant influence on changing the diameter, number of cut-outs and their arrangement on the deformation scale and the corresponding value of the critical force. Despite the variable parameters, each profile retained the same buckling form in the form of two half-waves. The obtained results allowed to observe that the increase in the cut-out diameter significantly effects the deterioration of the mechanical properties.

The second part of the analysis was devoted to the work of profiles after loss of stability. Numerical calculations were also performed in the Abaqus program. The aim of the analysis was to investigate profile behaviour in a post-critical state with the implemented lower buckling mode, which was determined in the first part of the tests, and to check whether the obtained characteristics are stable. During the loading of the structure a deepening form of laminate buckling was observed. In all analysed cases, the scope of the profiles’ work was determined by the load initiating failure of the first composite layer, according to the Tsai-Wu failure criterion. Carried out analysis allowed for the determination of composite failure maps. Figure 8 shows the localisation where the initiation of damage of the first layer of the laminate may occur. For profiles with one row of holes on the web, the greatest risk of failure occurs at its connection with the flange (Figure 8a,b). The same situation is in the case with the non-perforated model (Figure 8j). The situation changes when a profile has more cut-outs. Due to making additional holes in the shelves, the place most exposed on failure is the area of the hole closest to the outer edge of the shelf (Figure 8c,d). In the case of models with two rows of holes made on the web, the failure will occur in the vicinity of one of them (Figure 8f–i).

Additionally, in Table 4, the numerical values of the load initiating the failure of the first layer of the composite material are summarized together with the number of the layer in which the achievement of the critical parameter 1 was identified.

The load value initiating the failure of the first layer is different for each model. On the basis of the results presented above, it can be concluded that the profile without cut-outs is the least susceptible to damage. However, the Z4 profile will deform at the lowest applied load, among all models. It is due to the location and diameter of the cut-outs on the web and shelves. In this case, failure of the first layer will occur when the load equals 4776 N, which indicates a value around 38% less than that required to load the model without cut-outs. Despite the significant difference between those two values, the Z4 profile retains good rigidity parameters.

The conducted analysis showed that the outer layers, on which the fibres are arranged at an angle of 0°, most often fail first. This situation is in 60% cases. The second most vulnerable layer for failure is layer 7, where fibres are arranged at an angle of 45°.

**Figure 9:**
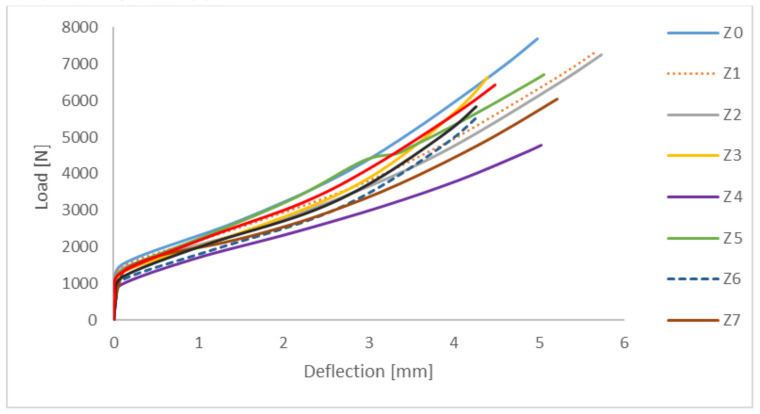
Post-critical equilibrium paths for a Z-section column using different perforations.

An additional post-failure analysis was performed by comparing the post-critical equilibrium paths (Figure 9).

The obtained characteristics have a stable nature of work, which confirm the structure’s ability to carry the load in the post-critical range. The determined post-critical equilibrium paths of compressed Z-section columns tend to decrease the stiffness of the structure due to the introduction of cut-outs on the shelves. Decrease in stiffness between the extreme profiles (profile without cut-outs and the profile with the lowest stiffness) amounts to approx. 37%. On the other hand, the decrease in stiffness for Z5 profile in relation to the profile without the cut-out is at the level of approx. 15%.

## 5. Conclusions

On the basis of the obtained results, it can be concluded that the diameter and arrangement of the cut-outs significantly affect on the critical state and postcritical behaviour of the tested structures. All models deformed in the asymmetrical two half-waves form. Critical forces and buckling modes have shown that the holes made in the profiles weaken the structure, but not as so much as to exclude them from the possibility of industrial application. The proper arrangement of the holes in the laminate allows achieving similar mechanical properties as for the non-perforated profile.

The holes located on the composite shelves have the least impact on the loss of stability of profile. The biggest difference in the value of the critical load in comparison with the non-perforated profile was noticed in the Z-profile with diameter of holes equal to 20 mm, located in the axis of the web and in the axis of the shelves. The difference in the value of the critical force in this case was around 30%. The remaining profiles showed a decrease in critical force of about 3–15% in compared to the laminate without holes. By changing the arrangement and diameter of the holes, can significantly affect the obtained results of the analysis as well as improve properties of element.

Based on the results of the nonlinear stability analysis, it can be noted that the profiles with one row of holes on the web are characterized by the failure of the first laminate layer at first, and the nevralgic area occurs at the connection of the web and the shelves. The situation changes when additional holes are added. More holes in the profile shelves causes failure of the first layer and the nevralgic place was located in the area of the opening located closest to the outer edge of the shelve. In the case of a profile with two rows of holes on the web, the failure will occur close to one of one of them. The deflection value of the analysed models also depends on the localisation of the holes. The difference in value between the two extreme profiles is approx. 0.16 mm.

The presented results of the numerical analysis confirm that thin-walled, perforated profiles with a Z-cross-section do not lose their stability in the post-critical range. In addition, perforated Z-profiles can be used as structural elements, and a well-chosen arrangement of the holes may prevent the mechanical properties from deteriorating.

From the results obtained, the following conclusions can be obtained:

- The presented results of the numerical analysis confirm that thin-walled, perforated profiles with a Z-cross-section do not lose their stability in the post-critical range.
- In the numerical tests, it was found that the profile with smaller size of holes located on the web causes less change in stiffness compared with the other configurations of holes.
- The perforated Z-profiles can be used as structural elements, and a well-chosen arrangement of the holes may prevent the mechanical properties from deteriorating.
- The performed analysis may be as the beginning of further research aimed at improving the obtained results by performing parametrisation of cut-outs and their arrangement, by changing the orientation of the fibre layers, as well as the type of profile cross-section.
- The problems related with failure analysis in the full load range seem worthy of further continuation.

## Figures and Tables

**Table 1:** Mechanical properties of carbon–epoxy composite.

Young’s Modulus		Shear Modulus	Poisson’s Coefficient	Tensile Strength		Shear Strength	Compression Strength	
E_1_ (0°) MPa	E_2_ (90°) MPa	G_1,2_ MPa	V_12_	F_TU1_ (0°) MPa	F_TU2_ (90°) MPa	F_SU_ (45°) MPa	F_CU1_ (0°) MPa	F_CU2_ (90°) MPa
143.53	5826	3845	0.36	2221	49	83.5	641	114

**Table 2:** Comparison of buckling load values.

Buckling Load [N] FEM Analysis	Bucklin Load [N] Experimental	Differences [%]
2189	2168	0.96

**Table 3:** The values of critical forces for all tested cases.

Profile	Z0	Z1	Z2	Z3	Z4	Z5	Z6	Z7	Z8	Z9
Critical Force [N]	2189.3	2125	1965.8	1907.8	1528.7	2039.4	1698.3	1863.4	1863.2	2026.7

**Table 4:** The value of the load initiating the failure of the first layer, along with its number and the percent of decrease in the stiffness depending on the Z0 column.

Profile	The Load Value Initiating the Failure of the First Layer [N]	Decrease in the Stiffness Depending on the Z0 Column [%]	The Number of the Failure Layer
Z0	7680		8
Z1	7344	4.38	7
Z2	7260	5.47	7
Z3	6636	13.59	1
Z4	4776	37.81	7
Z5	6720	12.5	8
Z6	5520	28.13	1
Z7	6036	21.41	7
Z8	5832	24.0	8
Z9	6420	16.41	8

## Data Availability

Data is contained within the article.
